# Age-dependent increase of treatment-related mortality in older patients with aggressive B cell lymphoma: analysis of outcome, treatment feasibility, and toxicity in 1171 elderly patients with aggressive B cell lymphoma—data from phase II and III trials of the DSHNHL (German High-Grade Non-Hodgkin’s Lymphoma Study Group)

**DOI:** 10.1007/s00277-020-04345-3

**Published:** 2020-11-26

**Authors:** Florian Zettl, Marita Ziepert, Bettina Altmann, Samira Zeynalova, Gerhard Held, Viola Pöschel, Karin Hohloch, Gerald G. Wulf, Bertram Glass, Norbert Schmitz, Markus Loeffler, Lorenz Trümper

**Affiliations:** 1 Department of Hematology, Oncology and Palliative Care, Klinikum Traunstein, Traunstein, Germany; 2 Institute for Medical Informatics, Statistics and Epidemiology, University of Leipzig, Leipzig, Germany; 3 Department of Internal Medicine, University Hospital Saarland, Homburg, Germany; 4 Department of Hematology and Medical Oncology, Georg August University Göttingen, Göttingen, Germany; 5 Hematology and Oncology, Kantonsspital Graubünden, Chur, Switzerland; 6 Department of Hematology, Oncology, and Tumor Immunology, Helios Klinikum Berlin-Buch, Berlin, Germany; 7 Department of Internal Medicine A, University of Münster, Münster, Germany

**Keywords:** Aggressive lymphoma, Diffuse large cell lymphoma, Elderly patients, Treatment-related mortality, Infections

## Abstract

In elderly patients (pts) with aggressive B cell lymphoma (aNHL), curative treatment often cannot be administered because of comorbidities and tolerability. We analyzed the influence of age in pts > 60 years receiving the R-CHOP-14 regimen within different prospective DSHNHL trials. Of the RICOVER-60 trial and CHOP-R-ESC trials, 1171 aNHL pts were included in this retrospective analysis of age-dependent event-free survival (EFS), progression-free survival (PFS), and overall survival (OS). All patients received prophylactic G-CSF, and anti-infective prophylaxis with amphotericin B mouth wash and oral fluorchinolone was optional. In the CHOP-R-ESC trials, prophylaxis was augmented to include mandatory continuous orally administered aciclovir and a pneumocystis prophylaxis with cotrimoxazole as well as oral fluorchinolones during neutropenia. The patient population was separated into 4 age groups (61–65 years, 66–70 years, 71–75 years, and 76–80 years). The results from the RICOVER-60 trial were subsequently confirmed in the following CHOP-R-ESC trials by a multivariate analysis adjusted for IPI factors and gender. Significant differences (*p* < 0.001) in EFS, PFS, and OS were seen between age groups (RICOVER-60). Hematotoxicity, infections, and TRM increased with age. TRM was significantly elevated in the age group 76–80 years. Therefore, this analysis shows that an age above 75 years defines an especially vulnerable patient population when being treated with chemoimmunotherapy for aNHL. Prophylactic anti-infective drugs are essential and clinically effective in reducing morbidity when treating elderly aNHL pts.

**Supplementary Information:**

The online version contains supplementary material available at 10.1007/s00277-020-04345-3.

## Introduction

Aggressive B cell lymphomas (aNHL) are a heterogeneous group of aggressive B cell neoplasms including diffuse large B cell lymphoma (DLBCL), the most frequent lymphoid neoplasia accounting for one-third of all malignant lymphomas. R-CHOP (rituximab, cyclophosphamide, doxorubicin, vincristine, and prednisolone) is the current treatment standard, leading to high cure rates especially in younger patients [1]. The incidence of aNHL, however, increases with age, and almost 50% of patients are older than 60 years [2]. In older patients, prognosis is almost identical to that of younger individuals if patients can be treated with full-dose immunochemotherapy, as demonstrated in several large phase III trials [2, 3]. However, administration of fully dosed chemotherapy is limited by the age-dependent increase of comorbidities and higher treatment-related mortality (TRM) [4]. Dose reductions are frequent and correlate with an inferior prognosis [5]. Supportive measures such as growth factor support and anti-infective prophylaxis are recommended by the American Society of Clinical Oncology (ASCO) [6] in order to facilitate treatment at full dose and reduce toxicity. Here we analyzed the feasibility and toxicity of the R-CHOP-14 regimen within consecutive prospective DSHNHL phase II and III trials (RICOVER-60, DENSE-R-, SMARTE-R-, and SEXIE-R-CHOP-14) for patients between 60 and 80 years of age. We investigated the impact of age on the outcome, feasibility, and toxicity of lymphoma therapy by dividing the patient population into four age groups (61–65 years, 66–70 years, 71–75 years, and 76–80 years). Furthermore, we analyzed whether the implementation of a stringent anti-infective prophylaxis as implemented in the most recent trial populations is able to reduce the treatment-related mortality (TRM).

## Material and methods

### Patients and treatment

Since 2000, 1823 patients (pts) with aggressive B cell lymphoma between the age of 61 and 80 years were treated in DSHNHL (German High-Grade Non-Hodgkin’s Lymphoma Study Group) phase III trials RICOVER-60 and consecutive phase-II CHOP-R-ESC trials: DENSE-R-, SMARTE-R-, and SEXIE-R-CHOP-14. The RICOVER-60 trial was a four-armed randomized phase III trial analyzing the impact of 8 applications rituximab (R-CHOP-14 versus CHOP-14) as well as the impact of two additional cycles of CHOP-14 (6 versus 8 cycles) on the outcome of elderly patients with aggressive lymphoma [3]. A total of 1242 patients have been recruited into the RICOVER-60 trial of whom 620 patients were randomized to R-CHOP-14; 610 patients with not withdrawn informed consent were included in this analysis. The aim of the subsequent CHOP-R-ESC phase II trials was to optimize the rituximab application in combination with 6 cycles CHOP-14 chemotherapy in patients between the age of 61 and 80 years with newly diagnosed aggressive B-NHL. The results of these trials were compared with the data of the RICOVER-60 trial. In the first, the DENSE-R-CHOP-14 trial, patients received in total 12 applications of rituximab (days 0, 1, 4, 8, 15, 22, 29, 43, 57, 71, 85, and 99) with 4 additional infusions of rituximab within the first 3 weeks of 6 cycles of CHOP-14. The primary end point was the assessment of pharmacokinetics (in the first 20 patients) and the safety of this rituximab application scheme [7]. In the second, the SMARTE-R-trial, patients received 8 cycles of rituximab; however, patients received a loading dose of R and were exposed to rituximab for a longer period of time (on days 4, 0, 10, 29, 57, 99, 155, and 239). The primary endpoint was the evaluation of the pharmacokinetics, safety, and toxicity of this extended rituximab therapy [8]. The third, the SEXIE-R-CHOP-14 trial, evaluated the role of 8 applications of rituximab with dose escalation in male patients (500 mg/m^2^ rituximab) to overcome the poorer prognosis of elderly male patients compared to their female counterparts. The primary objective of the SEXIE-R trial was the evaluation of the pharmacokinetics, safety, and toxicity of the upfront dose-dense rituximab schedule and the increased dose of 500 mg/m2 for elderly males [9]. All patients were to receive G-CSF from days 4 to 13 and an anti-infective prophylaxis with amphotericin B mouth wash starting at day 7 of each cycle until recovery of leukocytes > 1000/μl; oral fluorchinolone prophylaxis was optional. After a safety analysis at the time when 20 patients had been included into the DENSE-R-study had shown increased toxicity, prophylaxis in the CHOP-R-ESC trials was augmented to include mandatory oral fluorchinolones (starting day 6 of each cycle until recovery of leukocytes > 1000/μl), oral aciclovir (4 × 400 mg/day), and pneumocystis prophylaxis with cotrimoxazole (960 mg, 2 tablets on 2 consecutive days per week). We finally included 1171 pts with aggressive NHL receiving rituximab-based chemoimmunotherapy in the RICOVER-60 (*n* = 610), the DENSE-R- (*n* = 104/124 the first 20 patients without mandatory anti-infective prophylaxis were excluded), SMARTE-R- (*n* = 189), and the SEXIE-R-CHOP-14 trial (*n* = 268) into an analysis of event-free survival (EFS), progression-free survival (PFS), and overall survival (OS). TRM and the impact of augmented anti-infective prophylaxis, established within the CHOP-R-ESC trials, were investigated in comparison to the RICOVER-60 trial. We subdivided the population into 4 age groups (61–65 years, 66–70 years, 71–75 years, and 76–80 years) to analyze the outcome, feasibility, toxicity, and the effects of prophylaxis in an age-dependent manner.

### Statistical analysis

Event-free survival (EFS) as the primary endpoint of the RICOVER-60/CHOP-R-ESC trials was defined as the time from randomization/registration to disease progression, start of salvage treatment, additional (unplanned) treatments, relapse, or death from any cause. Progression-free survival (PFS) was defined as time from randomization/registration to disease progression, relapse, or death from any cause. Overall survival (OS) was defined as the time from randomization/registration to death from any cause. EFS, PFS, and OS were estimated according to Kaplan and Meier [10].

The separation of patients into the 4 age groups (61–65 years, 66–70 years, 71–75 years, and 76–80 years) resulted from a martingale residual analyses. Within the four age groups, univariable outcome analyses were performed, and log-rank tests are presented. Proportional hazard models for the four age groups were adjusted for the factors of the International Prognostic Index (IPI, i.e., lactate dehydrogenase (LDH) > normal, ECOG > 1, stages III/IV, and extranodal involvement > 1) and gender. Hazard ratios (HR) with 95% confidence intervals (CI) and *p* values are presented. For comparison of age groups regarding patient characteristics, infections CTC grades 3/4 and treatment-related mortality (TRM) chi-square and, if necessary, Fisher’s exact tests were used. For the estimation of the relative dose of doxorubicin (representing the protocol adherence of CHOP therapy), the body surface area (BSA) was assessed according to DuBois et al. [11] for each of the cycles. With these values, we assessed for all patients the absolute dose of doxorubicin (mg) given per BSA (m^2^) for each cycle. The relative dose of doxorubicin was then calculated as the sum of given doses (mg/m^2^) over the cycles divided by the planned dose for 6 (6 × 50 mg/m^2^) cycles according to RICOVER-60/CHOP-R-ESC protocol. Curves of relative dose were estimated according to Kaplan and Meier technique [12].

The significance level was set at *p* = 0.05 (two-sided). Statistical analyses were done with IBM SPSS Statistics 22 software (SPSS, Chicago, IL).

## Results

**Fig. 1:**
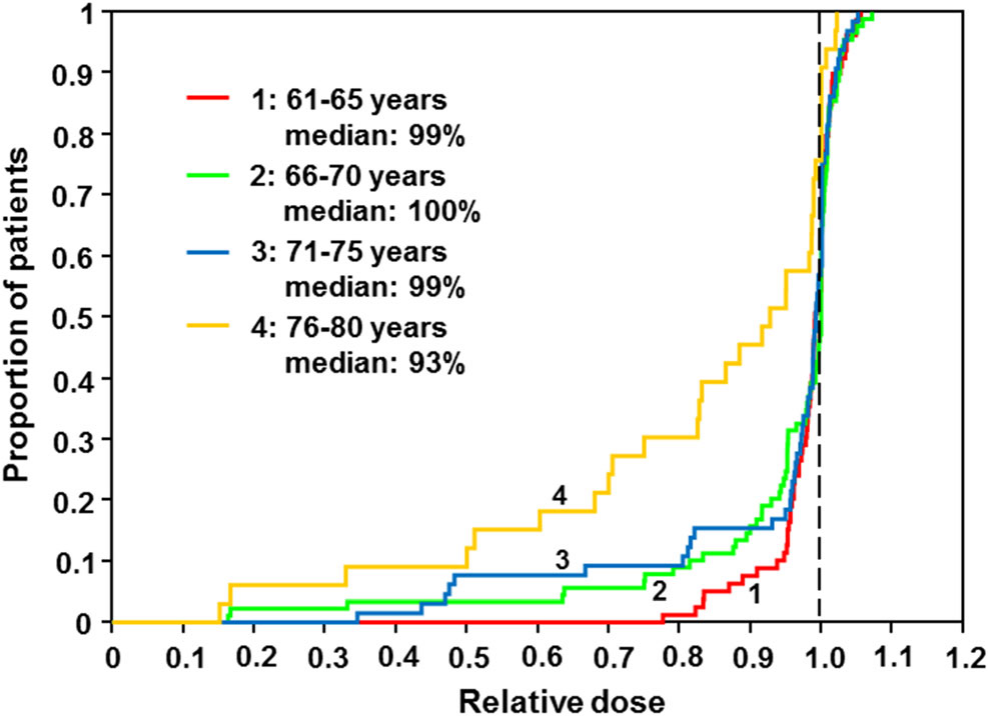
Relative dose doxorubicin RICOVER-60 study—age groups patients 61–80 years, 6-CHOP-14 + 8xR (*n* = 306)

**Fig. 2:**
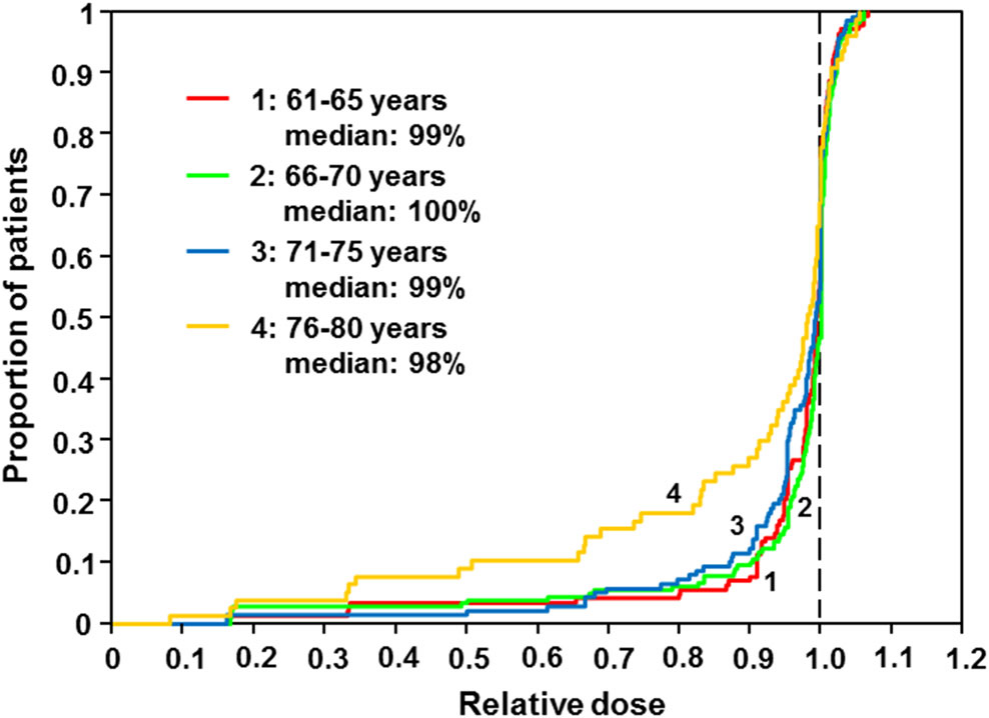
Relative dose doxorubicin CHOP-R-ESC study—age groups patients 61–80 years (*n* = 561)

**Fig. 3:**
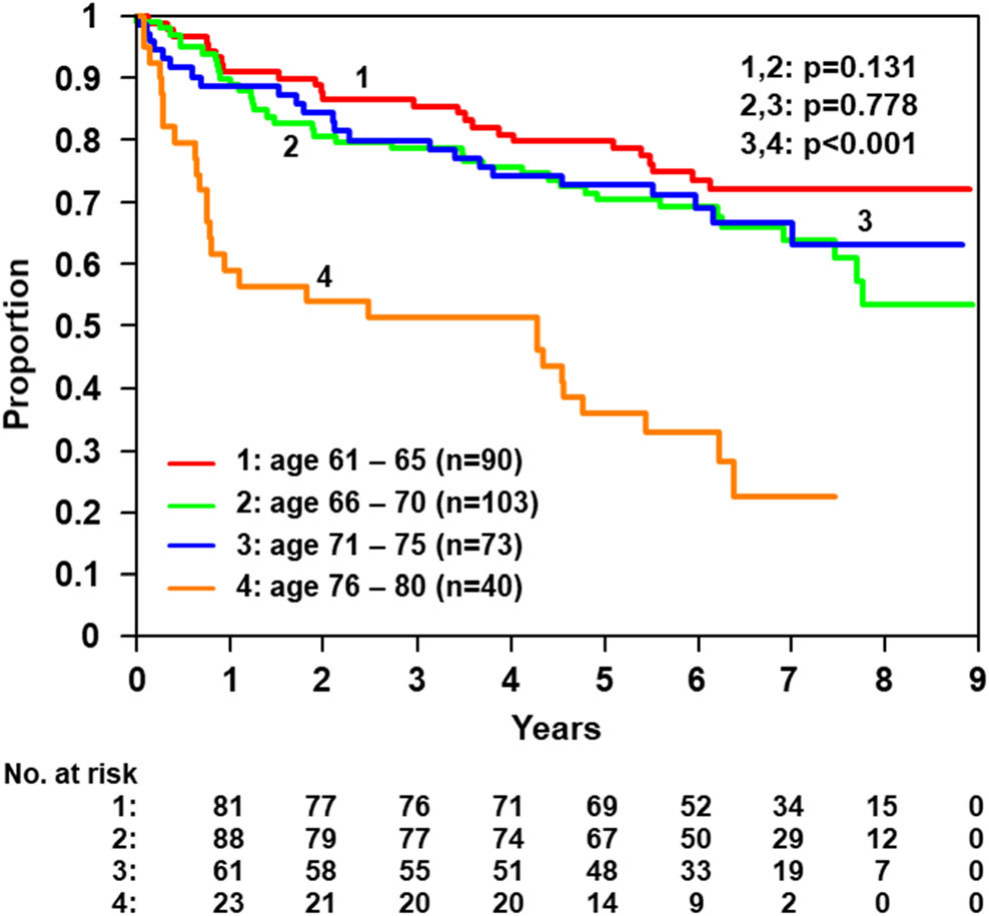
OS according to age group RICOVER-60 study—age groups patients 61–80 years, 6-CHOP-14 + 8xR (*n* = 306)

**Fig. 4:**
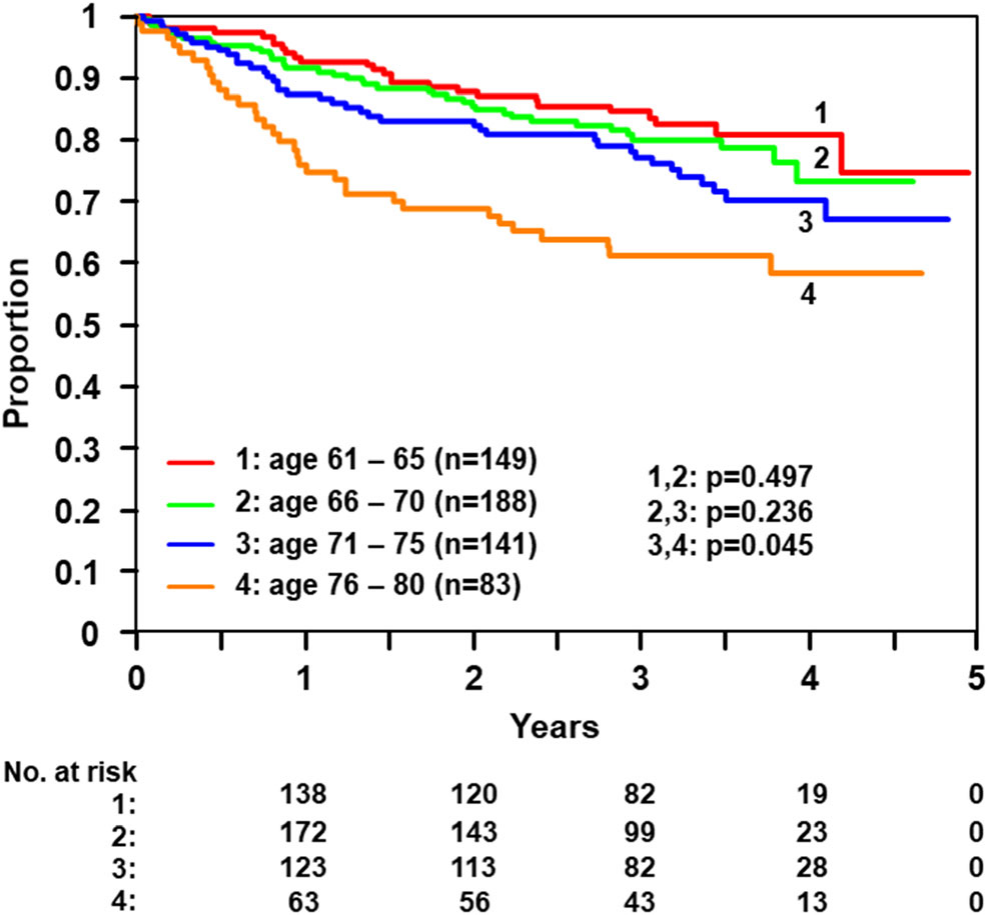
OS according to age group CHOP-R-ESC trials—age groups patients 61–80 years (*n* = 561)

In 1144 of 1171 patients (98%), a reference pathological diagnosis was available, and 946 of 1144 patients were diagnosed with DLBCL (83%). The clinical characteristics and IPI risk factors of the four age groups are shown in Tables 1 and 2 and the supplement Table 1. Subdividing the two study cohorts RICOVER-60 and CHOP-R-ESC into the four age groups within RICOVER-60 trial (6/8-CHOP-14 + 8xR) revealed significantly increased numbers of female patients (*p* < 0.001), more elevated LDH values (*p* = 0.003), and worse ECOG > 1 performance scores (*p* = 0.001) in elderly age groups (supplement Table 1) and within CHOP-R-ESC trials significantly more female patients (*p* = 0.004) and a trend to worse ECOG > 1 performance scores (*p* = 0.097) for the patients from elderly age groups (Table 2). Dose reductions were most frequent in the age group 76–80 years, however, to a smaller extent in the CHOP-R-ESC trial (Fig. 1 and Fig. 2); up to 70% of these pts from RICOVER-60 trial did not receive the complete chemotherapy as planned (Table 4). Hematotoxicity, infections, and TRM increased with age. TRM was significantly higher (*p* = 0.029) for the age group 76–80 years (amounting to 11% in the CHOP-R-ESC trials) as compared to 4% for the age group 71–75 years (Tables 3 and 4). In comparison, in the RICOVER-60 trial, TRM for 6-CHOP-14 + 8xR was 20% and 8%, respectively (*p* = 0.081). Higher TRM (but not higher mortality due to lymphoma) resulted in significant inferior EFS, PFS, and OS in the age group 76–80 years as compared to patients aged 71–75 years in both trials (RICOVER-60 3 years EFS, 44% (95% CI: 28–59) versus 68% (95% CI: 56–78) *p* < 0.001; PFS, 49% (95% CI: 33–64) versus 78% (95% CI: 68–88) *p* < 0.001; OS, 51% (95% CI: 36–67) versus 80% (95% CI: 70–89) *p* < 0.001 (Table 3, Fig. 3); R-CHOP-ESC 3-years EFS, 53% (95% CI: 42–64) versus 71% (95% CI: 63–78) *p* = 0.011; PFS, 57% (95% CI: 46–68) versus 74% (95% CI: 67–81) *p* = 0.011; OS, 61% (95% CI: 50–72) versus 77% (95% CI: 70–84) *p* = 0.045) (Table 3, Fig. 4). Multivariate analysis of the four age groups adjusted for IPI-factors and gender showed similar results with significant hazard ratios (HR) only between age groups 76–80 years and 71–75 years (RICOVER-60: EFS, HR = 1.9 (95% CI: 1.1–3.1) *p* = 0.020; PFS, HR = 2.3 (95% CI: 1.3–4.1) *p* = 0.004; OS, HR = 2.2 (95% CI: 1.3–4.0) *p* = 0.006; CHOP-R-ESC: EFS, HR = 1.7 (95% CI: 1.1–2.7) *p* = 0.013; PFS, HR = 1.8 (95% CI: 1.1–2.8) *p* = 0.011; OS, HR = 1.6 (95% CI: 1.0–2.5) *p* = 0.051).

**Table 1:** RICOVER-60 study—age groups patients 61–80 years, 6-CHOP-14 + 8xR (*n* = 306) demographics

	61–65 years	66–70 years	71–75 years	76–80 years
	*n* = 90	*n* = 103	*n* = 73	*n* = 40
Male	54 (60%)	59 (57%)	34 (47%)	21 (52%)
Female	36 (40%)	44 (43%)	39 (53%)	19 (48%)
Age, median (range)	62 (61, 65)	68 (66, 70)	73 (71, 75)	78 (76, 80)
LDH > UNV	39 (43%)	45 (44%)	37 (51%)	31 (78%)
ECOG > 1	13 (14%)	10 (10%)	9 (12%)	11 (28%)
Stage III/IV	52 (58%)	50 (48%)	26 (36%)	24 (60%)
Extranod. involvement > 1	17 (19%)	21 (20%)	6 (8%)	8 (20%)
IPI 1	29 (32%)	34 (33%)	26 (36%)	5 (12%)
2	24 (27%)	32 (31%)	23 (32%)	10 (25%)
3	22 (24%)	24 (23%)	19 (26%)	13 (32%)
4, 5	15 (17%)	13 (13%)	5 (7%)	12 (30%)
Extranod. involvem.	40 (44%)	59 (57%)	43 (59%)	19 (48%)
Bulky disease	42 (47%)	36 (35%)	22 (30%)	17 (42%)
B symptoms	36 (40%)	26 (25%)	23 (32%)	13 (32%)
BM involvement	4 (4%)	5 (5%)	2 (3%)	3 (8%)

**Table 2:** CHOP-R-ESC trials—age groups patients 61–80 years (*n* = 561) demographics

	61–65 years	66–70 years	71–75 years	76–80 years
	*n* = 149	*n* = 188	*n* = 141	*n* = 83
Male	94 (63%)	103 (55%)	74 (52%)	32 (39%)
Female	55 (37%)	85 (45%)	67 (48%)	51 (61%)
Age, median (range)	63 (61, 65)	68 (66, 70)	73 (71, 75)	77 (76, 80)
LDH > UNV	78 (52%)	99 (53%)	73 (52%)	47 (57%)
ECOG > 1	12 (8%)	25 (13%)	17 (12%)	16 (19%)
Stage III/ IV	95 (64%)	112 (60%)	84 (60%)	52 (63%)
Extranod. involvement > 1	48 (32%)	57 (30%)	42 (30%)	23 (28%)
IPI 1	37 (25%)	47 (25%)	36 (26%)	16 (19%)
2	32 (22%)	45 (24%)	34 (24%)	24 (29%)
3	49 (33%)	51 (27%)	40 (28%)	20 (24%)
4, 5	31 (21%)	45 (24%)	31 (22%)	23 (28%)
Extranod. involvem.	99 (66%)	123 (65%)	99 (70%)	47 (57%)
Bulky disease	52 (35%)	67 (36%)	52 (37%)	31 (37%)
B symptoms	42 (28%)	63 (34%)	41 (29%)	21 (25%)
BM involvement	13 (9%)	17 (9%)	13 (9%)	3 (4%)

**Table 3:** Comparison of survival data, treatment-related mortality, and rate of infections between the age groups in the RICOVER-60 and CHOP-R-ESC trials

	Age	EFS	PFS	OS	TRM	Infections
	Years	3-year rate				CTC
						grade 3,4
RICOVER-60 trial 6xCHOP-14 + 8R (*n* = 306)						
*n* = 90	61-65	72%	78%	85%	0%	24%
*n* = 103	66-70	70%	72%	79%	3%	20%
*n* = 73	71-75	68%	78%	80%	8%	33%
*n* = 40	76-80	44%	49%	51%	20%	44%
*p* values (all age groups)		*p* < 0.001	*p* < 0.001	*p* < 0.001	*p* < 0.001	*p* = 0.025
*p* values (76–80 vs. 71–75 years)		*p* < 0.001	*p* < 0.001	*p* < 0.001	*p* = 0.081	*p* = 0.263
CHOP-R-ESC trials (*n* = 561)						
*n* = 149	61-65	74%	78%	85%	1%	10%
*n* = 188	66-70	68%	75%	80%	4%	18%
*n* = 141	71-75	71%	74%	77%	4%	24%
*n* = 83	76-80	55%	57%	61%	11%	28%
*p* values (all age groups)		*p* = 0.025	*p* = 0.007	*p* = 0.001	*p* = 0.011	*p* = 0.001
*p* values (76–80 vs. 71–75 years)		*p* = 0.011	*p* = 0.011	*p* = 0.045	*p* = 0.029	*p* = 0.501

**Table 4:** Course of chemotherapy according to age groups for 6/8-CHOP-14 + 8xR (RICOVER-60 trial) and CHOP-R-ESC trials

Course of chemotherapy		% of age group				Total
		61–65 years	66–70 years	71–75 years	76–80 years	
RICOVER-60 trial 6-CHOP-14 + 8xR	*n*	90	103	73	40	306
	Regular	82 (91%)	92 (89%)	56 (77%)	26 (65%)	256 (84%)
	Non-regular because of toxicity	2 (2%)	7 (7%)	10 (14%)	13 (33%)	32 (11%)
RICOVER-60 trial 8-CHOP-14 + 8xR	*n*	108	85	71	40	304
	Regular	83 (77%)	54 (64%)	42 (59%)	12 (30%)	191 (63%)
	Non-regular because of toxicity	17 (16%)	18 (21%)	17 (24%)	22 (55%)	74 (24%)
CHOP-R-ESC trials	*n*	149	188	141	83	561
	Regular	138 (93%)	173 (92%)	129 (92%)	66 (80%)	506 (90%)
	non-regular because of toxicity	4 (3%)	10 (5%)	9 (6%)	14 (17%)	37 (7%)

Remark: Regular course of chemotherapy means, patient received all planned cycles of chemotherapy. Non-regular course of chemotherapy because of toxicity means, patient received not all planned cycles of chemotherapy due to toxicity

Because of increased infectious complications seen in the first 20 patients included in the DENSE-R-trial, the DSHNHL introduced a stringent anti-infective prophylaxis for all subsequent patients treated in all CHOP-R-ESC trials. Adherence to this schedule was almost complete (61–65 years, 93%; 66–70 years, 92%; 71–75 years. 89%; and 76–80 years, 91%) compared to the much lower percentage of patients receiving prophylaxis in the preceding RICOVER-60 trial (6xCHOP-14 + 8xR: 33%, 36%, 33%, 41%) resulting in higher numbers of patients being treated with interventional antibiotics (49%, 52%, 59%, 53%) compared to a much lower number of patients in the R-CHOP-ESC trials (26%, 31%, 36%, and 48%). The rate of grades 3 and 4 infections per patient was also different in these two patient cohorts with significantly lower numbers in the R-CHOP-ESC trials (19%) in comparison to the RICOVER-60 trial for 6xCHOP-14 + 8xR (28%; *p* = 0.005) (data within age groups: RICOVER-60 6xCHOP-14 + 8xR, 24%, 20%, 33%, 44%; R-CHOP-ESC, 10%, 19%, 24%, 28%). The TRM was significant lower for the R-CHOP-ESC trials in comparison to the whole RICOVER-60 cohort (4% vs. 7%, *p* = 0.038). The TRM was reduced by almost 50% in the more recent R-CHOP-ESC trials compared to the original RICOVER-60 trial (for details see supplement Table 2).

## Discussion

Choosing the best treatment for elderly patients with aggressive lymphoma is a clinical challenge. The number of patients not treated at all still increases with age [13]. More frequently, the age-dependent increase of relevant comorbidities [14] leads to a substantial increase in patients receiving less than full-dose immunochemotherapy. On the other hand, several trials incorporating R-CHOP have shown that many elderly patients can be cured, depending, among others, on the dose and dose intensity of chemotherapeutic agents actually being administered [2, 3]. If the relative dose intensity decreases below 70%, the relapse rate increases dramatically [5, 15]. As treatment-related toxicity dramatically increases with age, measures to decrease treatment-related morbidity and mortality in elderly patients are urgently needed.

Our analysis from a series of prospective multicenter trials is based on the R-CHOP-14 regimen administered at full doses. It shows that beyond the age of 75 years, there is a striking rise in TRM. One might speculate that the usage of CHOP-14 (instead of CHOP-21) regimen is responsible for this increase. However, there are currently no data available supporting this hypothesis. On the contrary, Cunningham et al. included patients in the same age group (56% of pts. > 60 years, range 19 to 88 years) comparing R-CHOP-21 with R-CHOP-14 and demonstrated g a higher infection rate in patients treated with R-CHOP-21. [16] The cut-off point of 75 years is among others supported by a retrospective analysis based on the SEER database in which the age above 75 years was identified as an independent risk factor for death within the first 30 days of treatment [17]. Therefore, measures to reduce toxicity and TRM in the elderly including a stringent antibiotic prophylaxis are of great importance for patients treated with immunochemotherapy. This might also be relevant for patients treated with R-CHOP-21 as the higher infection rate in these patients is possibly due to a lower rate of G-CSF prophylaxis compared to the CHOP-14 trials. In patients between 76 and 80 years of age, we observed a cumulative TRM in the RICOVER-60 trial of 20%. TRM dropped to 11% in the subsequent CHOP-R-ESC trials of the DSHNHL, although these trials used either higher doses and/or more frequent administration (DENSE-R, SEXIE-R) or an extended exposure time of rituximab (SMARTE-R). In the absence of randomized data, we hypothesize that a main reason for this improvement might be the introduction of a stringent infectious prophylaxis with a > 90% adherence in the CHOP-R-ESC trials. We found a TRM reduction by almost 50% (Table 3) compared to the RICOVER-60 study. The cut-off for an increased TRM in patients over the age of 75 years remained the same through all trials analyzed. We conclude that optimal supportive measures like the administration of prophylactic antibiotics, oral amphotericin B, and virostatic agents enable the application of full-dose immunochemotherapy in larger fractions of elderly patients with less toxicity, leading to improved cure rates. Well-designed strategies to select individualized prophylactic measures based on comorbidities and the history of a given patient are urgently needed. Data from prospective randomized trials for the increasing number of patients over 80 years are still missing. A phase II trial with a dose-reduced R-CHOP (R-miniCHOP) showed promising results, with a 2-year-PFS of 47% and OS of 59% [18]. Very recently, the data of a phase II trial applying ofatumumab in combination with miniCHOP and a mandatory prephase treatment with vincristine and prednisolone reported an improved 2 years OS of 64.7% [19]. The efficacy of bendamustine in elderly and frail patients is currently being analyzed. Park et al. published a smaller phase II trial with very modest results [20] and a median OS of 10.2 months and PFS of 5.4 months. The prospective B-R-ENDA phase II trial of the DSHNHL included pts over 80 years of age and pts being too frail to be included in the R-CHOP-ESC trials reported a 2-years OS of 42% [21]. Such studies and the results reported here highlight the clinical dilemma of treating elderly patients with aggressive lymphoma: efficacy is closely correlated with treatment intensity frequently resulting in high TRM. This analysis shows that a remarkable proportion of older patients, in particular those between 76–80 years, can receive treatment comparable to younger patients. This proportion might further increase by further optimizing supportive measures. Supportive measures (pre-phase treatment, close surveillance, prophylactic anti-infective therapy) are highly effective when treating elderly pts with aggressive lymphoma and result in significantly better treatment outcome without changing immunochemotherapy. The challenge remains to identify those elderly pts who will benefit from standard treatment if optimal prophylaxis is administered, but avoid such treatment and lower dose and dose intensity in those patients who will not benefit even if optimal prophylaxis is administered. Supporting clinical decision-making by wider and consequent use of geriatric assessment scores might help to improve the treatment results in older patients without changing state-of-the-art immunochemotherapy [15]. Recent studies trying to improve treatment results for elderly patients with DLBCL by adding new drugs to R-CHOP have largely failed, in part, because patients on these combinations suffered from more and previously unknown severe infections. Particularly in these situations, the addition of consequent anti-infective prophylaxis may help to make treatment more feasible and safe enabling patients to benefit from the superior anti-lymphoma effects offered by combining immunochemotherapy with new targeted therapies.

## Supporting information

ESM 1 (XLSX 10 kb). ESM 2 (XLSX 10 kb).
